# *The bench to community initiative*: community-based participatory research model for translating research discoveries into community solutions

**DOI:** 10.3389/fpubh.2024.1394069

**Published:** 2024-08-05

**Authors:** Jazma L. Tapia, Abigail Lopez, D. Bing Turner, Tonya Fairley, Tiah Tomlin-Harris, Maggie Hawkins, Pastor Rhonda Holbert, Lindsey S. Treviño, Dede K. Teteh-Brooks

**Affiliations:** ^1^Division of Health Equities, Department of Population Sciences, City of Hope Comprehensive Cancer Center, Duarte, CA, United States; ^2^Department of Health Sciences, Crean College of Health and Behavioral Sciences, Chapman University, Orange, CA, United States; ^3^Heritage Wellness Collective, Claremont, CA, United States; ^4^TS Fairley Hair Restoration Center, Covina, CA, United States; ^5^My Style Matters Inc., Atlanta, GA, United States; ^6^California State University Los Angeles, Los Angeles, CA, United States; ^7^Celebrate Life Cancer Ministry, Inglewood, CA, United States

**Keywords:** behavioral interventions, black people, implementation and dissemination science, community-based participatory research (CBPR), breast cancer, endocrine disrupting chemicals (EDC)

## Abstract

Community-based participatory research (CBPR) is an effective methodology for translating research findings from academia to community interventions. The Bench to Community Initiative (BCI), a CBPR program, builds on prior research to engage stakeholders across multiple disciplines with the goal of disseminating interventions to reduce breast cancer disparities and improve quality of life of Black communities.

**Methods:**

The BCI program was established to understand sociocultural determinants of personal care product use, evaluate the biological impact of endocrine disrupting chemicals, and develop community interventions. The three pillars of the program include research, outreach and engagement as well as advocacy activities. The research pillar of the BCI includes development of multidisciplinary partnerships to understand the sociocultural and biological determinants of harmful chemical (e.g., endocrine disrupting chemicals) exposures from personal care products and to implement community interventions. The outreach and engagement pillar includes education and translation of research into behavioral practice. The research conducted through the initiative provides the foundation for advocacy engagement with applicable community-based organizations. Essential to the mission of the BCI is the participation of community members and trainees from underrepresented backgrounds who are affected by breast cancer disparities.

**Results:**

Two behavioral interventions will be developed building on prior research on environmental exposures with the focus on personal care products including findings from the BCI. In person and virtual education activities include tabling at community events with do-it-yourself product demonstrations, *Salon Conversations*—a virtual platform used to bring awareness, education, and pilot behavior change interventions, biennial symposiums, and social media engagement. BCI’s community advisory board members support activities across the three pillars, while trainees participate in personal and professional activities that enhance their skills in research translation.

**Discussion:**

This paper highlights the three pillars of the BCI, lessons learned, testimonies from community advisory board members and trainees on the impact of the initiative, as well as BCI’s mission driven approaches to achieving health equity.

## 1 Introduction

The breast cancer mortality gap for Black women continue to widen when compared to other racial/ethnic groups (1). Black women are more likely to be diagnosed with breast cancer under the age of 40, and 41% more likely to die from the disease than White women. These disparities are multifactorial, and growing evidence supports a connection between breast cancer development/progression and exposure to harmful chemicals [e.g., endocrine-disrupting chemicals (EDCs)] found in hair and other personal care products (2, 3). Parabens are a class of EDCs commonly used as preservatives in personal care products, are estrogenic and thought to be linked to breast cancer risk (4). Black women are disproportionately exposed to higher levels of parabens as they commonly use hair and other personal care products in greater abundance compared to other racial/ethnic groups (5–7). Furthermore, studies have shown that increased product usage is associated with increased levels of parabens detected in biological samples, and parabens have been detected at higher levels in urine samples from Black women compared to White women (8–10). Collectively, current research suggests paraben exposure may contribute to known breast cancer disparities with high mortality rates linked to Black women diagnosed with breast cancer (4, 11, 12). Despite this, interventions are lacking to reduce these persistent disparities warranting the need for effective methods that improves health education, resources for reducing exposures, and additional research (13–15).

Community-based participatory research (CBPR) has been shown to be an effective methodology for translating research findings into community solutions. CBPR is known by many names including *participatory action research*, *community-based research,* and *community to bench model* (16, 17). Despite the variations in terminology, the principles of CBPR require collaboration or partnership between the community and the academy across the research continuum. Israel et al. defines CBPR as active involvement of community members from research conceptualization to dissemination of findings with a focus on reducing social and environmental inequities through interventions and policy change (18). Two examples of interventions to reduce EDC exposures from personal care products using CBPR strategies include (1) The Health and Environmental Research on Makeup of Salinas Adolescents (HERMOSA) intervention study—reducing endocrine disrupting chemical exposures in personal care products of Mexican American adolescent girls; and the (2) Reduced Xenoestrogen (REDUXE) intervention study—*in vivo* molecular impact of estrogenic chemicals within human breast tissue from women without a prior cancer diagnosis (19–21). The HERMOSA intervention study used a bidirectional form of learning between the researchers and the young women of the Salinas community. This strategy was achieved through collaboration with 15 high school students that were recruited to participate as youth researchers. This role was pivotal in all aspects of the HERMOSA study, and particularly in building a trusting relationship and connection with the participants. The REDUXE study research team collaborated with the community-based organization, Breast Cancer Over Time, to recruit volunteers for their study focused on people who used one or more personal care product (s) containing parabens and phthalates. The purpose of the study was to reduce daily exposure to xenoestrogens for 28 days, followed by collection of biological samples, and testing at the molecular and cellular levels.

The *Bench to Community Initiative* (BCI) embodies the principles of CBPR and provides a holistic approach to addressing research questions that are then used to develop community interventions. Different from the HERMOSA and REDUXE programs, BCI focuses primarily on Black women and Black breast cancer survivors, a vulnerable and understudied population in the “environmental injustice of beauty” research discipline (22). The BCI stakeholders use research, outreach, and engagement as well as advocacy strategies that includes engagement across multiple disciplines with the goal of disseminating interventions to reduce breast cancer disparities associated with exposure to harmful chemicals in personal care products.

## 2 Application of CBPR methods in project implementation

### 2.1 The origins of the bench to community initiative

**Figure 1:**
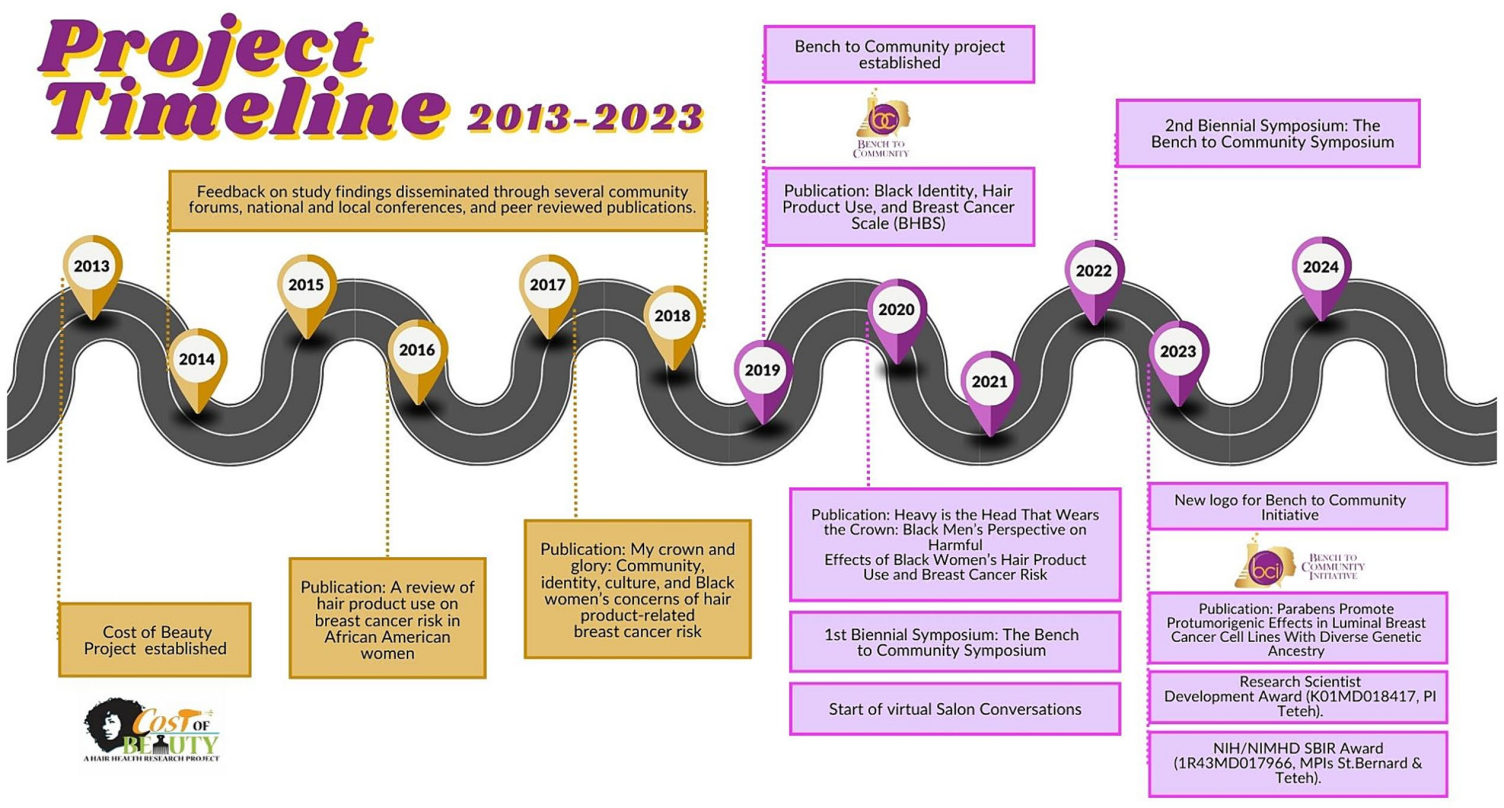
Historical timeline of the Bench to Community Initiative (BCI). The Cost of Beauty Project (2013) is the foundation for the inception of BCI, and feedback on the collaborative findings disseminated throughout the community has inspired the advancements of BCI present day (2024).

The BCI builds on findings from a CBPR project entitled *Is the Cost of Beauty Putting Black Women at Risk?* (COB), funded by the California Breast Cancer Research Program (23). The project was led by multiple principal investigators (MPIs) including two community-based organizations and an academic institution. The purpose of the project was to explore cultural factors related to hair product use and assess knowledge of EDC exposures, and associated breast cancer risk. Collectively, the main findings suggest hair and identity are affirming for Black women, there is lack of knowledge about EDCs in personal care products and breast cancer risk, there is resistance to selecting non-toxic products because “everything causes cancer,” and there is a need for culturally targeted health promotion interventions (24, 25). Additionally, the Black *identity, hair product use, and breast cancer scale* was developed (26). The scale is a valid measure for assessing sociocultural constructs associated with hair product use and perceived breast cancer risk of Black women. Feedback on study findings, which were disseminated through several community forums, 10 national and local conferences, and 4 peer reviewed publications, ignited a community education campaign about the association between EDCs and breast cancer risk. The community’s request for education, additional research, and interventions led to the formation of the BCI (see Figure 1 for historical timeline). BCI was initially a pilot research project funded by an internal American Cancer Society and foundation award with two co-principal investigators (Teteh-Brooks and Treviño) and supported by several community-based organizations. The initial research project was later expanded to an *initiative* due to continued research, outreach, and advocacy efforts directed by Teteh-Brooks, additional collaborators (e.g., St. Bernard, Hua, Tapia), and engagement with current community advisory board (CAB) members (manuscript co-authors).

## 3 The pillars of the BCI: research, community outreach and engagement, and advocacy

### 3.1 Research

The research pillar of BCI includes two overarching goals: (1) develop multidisciplinary partnerships with community stakeholders, scientists, and advocates to understand the sociocultural and biological determinants of harmful chemical (e.g., EDCs) exposures from personal care products and breast cancer risk; and (2) implement community interventions aimed at reducing breast cancer risk, including exposures from personal care products for Black people.

### 3.2 Community outreach and engagement

Through the community outreach and engagement pillar the aim is to educate and translate research into practice. Salon Conversations (SC), hosted by BCI CAB members, is a virtual platform used to bring awareness, education, and pilot behavior interventions. The symposium invites researchers, health advocates, breast cancer survivors or thrivers, community-based organizations, and media representatives to discuss and learn about EDCs and breast cancer risk. The SC forum is an extension of the biennial symposium, which includes a diverse representation of speakers and other stakeholders to bring awareness, educate, and provide strategies toward sustainable change related to breast health and reducing exposure to toxic chemicals found in personal care products. We use pre-and post-surveys to determine knowledge and assess motivations of individuals to reduce exposures to harmful chemicals contained in personal care products. In conjunction with social media and BCI’s website (27), SC serves as a mechanism for research implementation as it provides an outlet for continuous data collection, information dissemination and community engagement. Additionally, the use of social media platforms (i.e., Instagram, Facebook. Twitter/X, LinkedIn, and YouTube), and digital newsletters (Mailchimp) allow our collaborative to develop a unit of identity, thereby designing connections based on shared values, interests, and commitment to the overall mission of the initiative. The BCI team also engages community stakeholders via educational events (in-person and virtually) that promote breast health, and strategies for reducing exposures from harmful chemicals. In-person activities include do-it-yourself demonstrations using household ingredients like baking soda, coconut oil, and brown sugar to make toothpaste or body scrubs (28).

### 3.3 Advocacy

The research conducted through the initiative provides the foundation for advocacy engagement. Presently, we are developing strategies to intentionally engage in the legislative process and support efforts of community-based organizations focused on reducing harmful product exposures and breast cancer risk. CAB members received training from Shannon Lawrence, Founder of I Can Lead! to gain knowledge, skills, and strategies to effectively engage the community in legislative advocacy efforts (29). We also use our social media platforms to communicate policy announcements and updates to inform the community about ways they can engage in the legislative process.

## 4 BCI an example of CBPR modeling for translating research discoveries into community solutions

### 4.1 Research from the bench and community-based interventions

To understand the biological effects of parabens, Treviño’s laboratory including Tapia examined the effects of parabens on Black and White luminal breast cancer cell lines (11). They showed that paraben altered estrogen receptor target gene expression and cell viability was cell line specific. Particularly, in some cases, an increase in gene expression and cell viability was higher in the Black luminal breast cancer cell line than the White luminal breast cancer cell line.

Additionally, the first intervention from BCI will engage Black breast cancer survivors, funded by a National Institutes of Health (NIH)/National Institute on Minority Health and Health Disparities (NIMHD) Research Scientist Development Award (K01MD018417, PI Teteh-Brooks). The Black Breast Cancer Survivors’ Intervention (BBCSI) will be co-developed with community partners/CAB members from My Style Matters Inc. (Tomlin-Harris) and Celebrate Life Cancer Ministry (Holbert). In partnership with SOCAi Labs and funded by NIH/NIMHD, Fairley and Teteh-Brooks will support the development of a hair care recommendation application including safer product suggestions and health education for Black women (R43MD017966, MPIs St. Bernard, Teteh-Brooks).

### 4.2 Facilitation of collaborative and equitable partnerships through the integration of multidisciplinary perspectives

**Figure 2:**
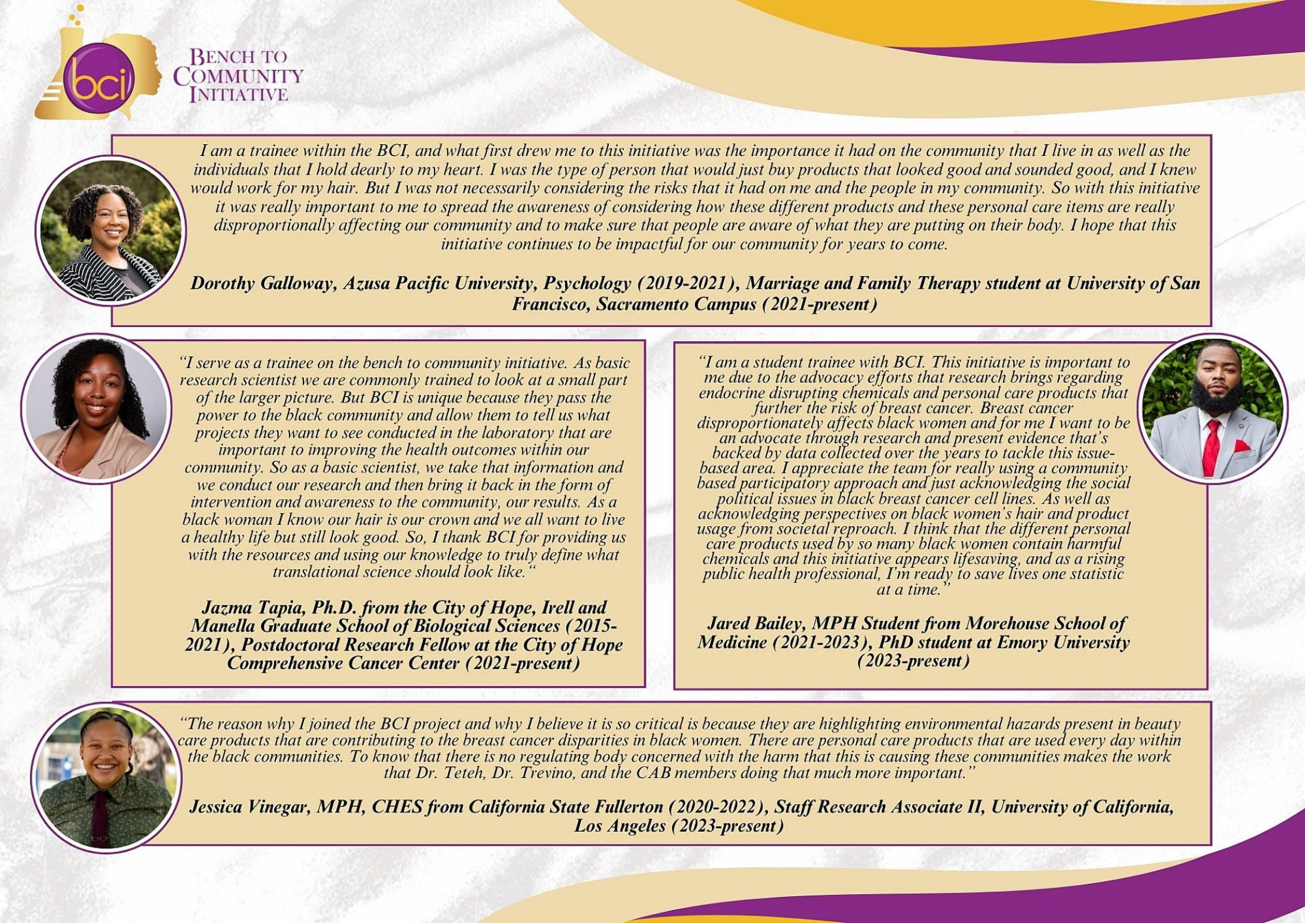
The Bench to Community Initiative (BCI) trainees share testimonies on the impact of the initiative. BCI provides opportunities for researchers of color at all academic levels (i.e., undergraduate, and graduate students as well as postdoctoral fellows). BCI trainee quotes were transcribed from our 2022 BCI Symposium video and include reflections from Dorothy Galloway (top), Dr. Jazma Tapia (middle left), Jared Bailey (middle right), and Jessica Vinegar (bottom).

BCI is a CBPR project composed of behavioral/intervention scientists, basic researchers, engineers, epidemiologists, and a CAB of stakeholders including Black breast cancer survivors, stylists, and public health experts. Use of the CBPR method allows partners to share decision-making and leadership responsibilities on culturally relevant public health issues (18). Models, such as BCI, structure sustainability by building and maintaining trust through constant collaboration with communities (16). The collaborative offers stakeholders opportunities to contribute to public health practice through quarterly meetings that deliver project updates, intervention, and dissemination discussions, and training relevant to CAB members’ positionality and knowledge deficits. These trainings are provided by researchers, consultants, and occasionally student trainees. Instructions by public health and basic science trainees provide opportunities to enhance their preparation for disseminating data at community forums, and local and national conferences. In addition to these opportunities, BCI provides mentoring and career development for researchers of color. To date, the BCI collaborative has mentored eight trainees from the undergraduate, graduate, and postdoctoral levels. Common themes from BCI trainee testimonies on the impact of the initiative include advancement of their research or career goals, the importance of acknowledging the needs of under-resourced communities and gaining knowledge on translating research findings through awareness, education, and advocacy. Complete trainee testimonies as shown in Figure 2 can also be accessed on the BCI YouTube channel from our 2022 Community Symposium (30).

### 4.3 Community partners’ perspectives on BCI collaboration and community impact

A Community Advisory Board or a CAB is a collective of community stakeholders or leaders who provide concerns and priorities for research agendas and support the implementation of these initiatives (31). BCI’s CAB has evolved since its initiation. This evolution is in part due to identifying mutually beneficial partnerships with shared interests, culture, and survivorship experiences. These community-academic partnerships foster trust and require all members to invest time in co-learning opportunities to achieve research goals. Phase 1 involved a 6-member CAB for the BCI which included a breast cancer survivor, public health advocate, hair stylist/trichologists, dermatologist, and hair product business owners. Each member was interviewed for the position and their roles and responsibilities were verbally discussed but not documented. This initial group met in person in 2020 prior to the COVID-19 pandemic shutdown to discuss the implementation of the research project funded by internal grant from the American Cancer Society. After the initial meeting, there were challenges with communication of research implementation goals because of the collective trauma of the COVID-19 pandemic, racial unrest, and competing responsibilities. As a result, four members of the phase 1 CAB (dermatologist, 1 business owner, 1 stylist, and public health advocate) resigned from their positions. The remaining CAB members (breast cancer advocate, stylist/trichologist/business owner) supported Teteh-Brooks in recruiting the next iteration of the CAB membership (phase 2).

In 2021, the structure of the CAB was modified to include written agreements that were signed on a yearly basis as well as interviews and approval of CAB positions by current members. Teteh-Brooks and the remaining CAB members recruited four members including additional breast cancer survivor, two public health advocates and a community-based organization director with similar goals as the BCI. Each CAB member served a 1-year term which was renewed in January of each year through a signed agreement. CAB members also received a modest $300 honorarium for attending 4-quarter meetings throughout the year. In 2022, three CAB members renewed their agreement and one resigned due to increasing personal and professional responsibilities. The CAB members were replaced through the process described previously. In phase 3, we currently have a 5-member BCI CAB which includes breast cancer survivors (3), public health advocates (2), hair product business/stylist/trichologist (1) and representation from three community-based organizations (CBOs, 3).

**Figure 3:**
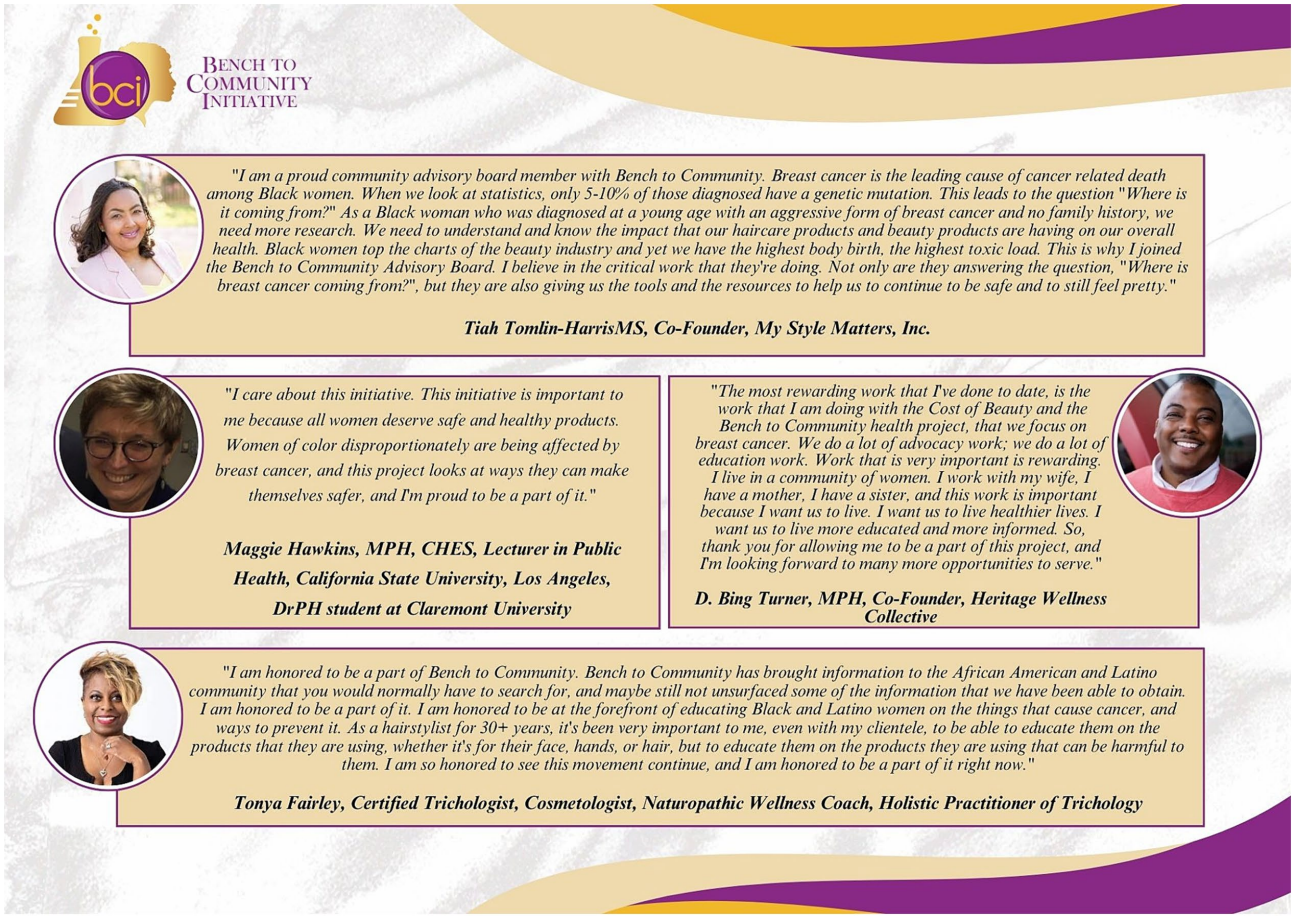
The Bench to Community Initiative (BCI) Community Advisory Board Members (CAB) share testimonies on joining the initiative and BCI’s impact on the community. Community stakeholders positioned on the BCI CAB shared common themes on why they joined the initiative in video format during BCI’s 2022 Community Symposium. Reflections from CAB members include Tiah Tomlin-Harris (top), Maggie Hawkins (middle left), D. Bing Turner (middle right) and Tonya Fairley (bottom).

Visibility of BCI is in part due to the commitment of community partners. Collaboration between the academy and community presents a unique opportunity to educate the public on the impact of EDCs on health with a focus on breast cancer risk and Black women. In their words, as shown in Figure 3 are testimonies of the current BCI CAB on joining the initiative and the impact of the project on the community. Common themes from the CAB’s testimonies include having the opportunity to educate the community on the impact of hair and other personal care product use on overall health outcomes. Additionally, CAB members convey their research contributions focused on reducing adverse exposures and acknowledge the work they have completed through the initiative as rewarding. The quotes were transcribed from videos of CAB members from BCI’s 2022 Community Symposium—an extended version of the SC program—and the recordings can also be found on our YouTube channel (32). In preparation for this paper submission, CAB members revised the manuscript during our in-person August 2023 retreat at the Shepherd’s Manor. The Shepherd’s Manor is a wellness center and intervention program for cancer survivors and caregivers founded by CAB member Pastor Rhonda Holbert (33).

### 4.4 Community outreach and engagement

The BCI collaborative’s Salon Conversations and biennial symposiums to date have supported the education of 516 participants (1,131 registrants, 46% attendance rate, as of June 2024) on harmful chemical exposures (e.g., EDCs) and breast cancer risk. The BCI social media and listserv accounts (Instagram, Facebook, Twitter, YouTube, LinkedIn, Mailchimp) have over 2,200 followers and subscribers combined (as of June 2024). Having a digital presence allows for contact with the community to provide safer beauty resources, research updates, increase awareness about toxic chemicals, and engage individuals in advocacy efforts. Additionally, the BCI website (https://www.bench2community.org/) serves as an information hub with details about the project’s aims, goals, and progress. The website also houses a resource list, mobile apps for safer products, and articles on EDCs and breast cancer risk. Between 2022 and 2023, the website was visited by over 16,000 unique visitors from the United States, South Africa, Philippines, Jamaica, United Kingdom, Canada, Mexico, Barbados, and Trinidad and Tobago. BCI also maintains an in-person presence in the community by partnering with local organizations to attend community educational events to facilitate do-it-yourself safer product demos and share resources on how to reduce exposure of harmful chemicals at home. In 2023, the research team participated in six community educational events and completed over 230 do-it-yourself demonstrations.

## 5 Discussion on challenges and lessons learned from the BCI

There is a growing movement in health disparities and translational research, which may be impacting the decline in cancer mortality rates for breast and other cancers (34, 35). Despite these declines, the gap between Black women and other racial/ethnic groups for breast cancer mortality rates continue to widen (36). While the research on health disparities has grown since the publication of the Report of the Secretary’s Task Force on Black and Minority Health (13, 37), the gaps in translation of health research into interventions is approximately 15–17 years (17, 38, 39). This time lag is in part due to the various phases of research activities (i.e., grant submissions, observational data collection, publication of findings) and behavioral intervention development [i.e., NIH Stage Model for Behavioral Intervention Development (40)] that must be achieved prior to implementation and dissemination of interventions. The BCI program aims to reduce these limitations by developing sustainable partnerships rooted in community concerns and collaboration with stakeholders to address health inequities through research, outreach & engagement, and advocacy. Through these partnership dynamics, community stakeholders and researchers alike engage to co-create interventions that promote health equity among under resourced communities. The following lessons since the inception of BCI, may provide context on how community stakeholders and academic organizations can collaborate to promote health equity for Black communities.

### 5.1 Invest time and resources to develop sustainable relationships with community advisory board (CAB) members and other community stakeholders

The process of building a sustainable CAB to support research initiatives has challenges rooted in human behavior. Academic and community partners must be realistic about their roles and responsibilities as flexibility and accountability is required for maintenance. Additionally, by developing a CAB with leaders of CBOs, the BCI created shared learning community resources and dissemination hub of research opportunities and findings. For example, BCI partnered with CAB member, Tiah Tomlin-Harris (founder of My Style Matters, Inc.), to host the Jane Doe Films and Entertainment One “Not So Pretty” watch party, directed by Kirby Dick and Amy Ziering, and produced by Alexandra Marske and Kat Nguyen (41). This four-part docuseries described the harmful consequences of toxic chemicals from personal care products. The partnership diversified the BCI audience to include communities in Atlanta, Georgia and promoted a co-leadership model for community education. Additionally, continuously fostering relationships with community partners has led to increased engagement of community education programs across multiple states. While CAB members are instrumental to the success of the BCI, the relationship is mutually beneficial. In addition to the yearly agreements, CAB members co-facilitate community education presentations (some compensated), are collaborators/contractors on local and federal grants, and have access to resources through academic institutions.

### 5.2 Availability of funding mechanisms to support multidisciplinary community-led research initiatives

The primary goal of the BCI is to implement community-led interventions. Funding to support this goal in designing and assessing these large-scale community clinical trials is limited (42). The initial funding structure of the BCI included institutional grant awards and research start-up from early-stage investigators. Grant proposals that included multidisciplinary aims submitted to foundations and federal mechanisms were not funded. There were challenges with convincing reviewers of the project scope as study sections or equivalent review structures are siloed (focused on one discipline expertise) (43). Each investigator as a result submitted independent project aims focused on laboratory, observational research, and intervention development. The data obtained from these independent projects will be incorporated in the community-led interventions.

The lack of diversity of funding mechanisms to support multidisciplinary projects can be a factor in the retention of participants, CAB, and sustainability of community-led initiatives. Community members deserve to be compensated for their time and knowledge when supporting research initiatives. Equitable compensation is often a challenge because of the funding limitations described previously. How much is enough to pay community members? The fair-market value calculator developed by the National Health Council is a tool to support community and academics in making equitable compensation decisions (44). Some solutions that were implemented between 2020 and 2023 to compensate CAB members included requesting speaking fees for education sessions from for-profit institutions, payment for recruitment fees for research projects to CBO members, adding CBO members to grant applications as contractors/consultants. Technical assistance, visibility, and trainings to support CAB members roles are non-monetary benefits that have also been provided.

Under resourced communities are often excluded from translational research implementation resulting in potentially detrimental health outcomes (45). Federal funding agencies have yet to understand the benefit of the CBPR or behavioral intervention methods. Yet, sustaining a relationship with the community requires prolonged funding that produce interventions (46). While the NIH prioritizes traditional translational research, more work is needed to secure funding for CBPR models to successfully promote healthier communities (42).

### 5.3 The taxation of minoritized community stakeholders and early-stage investigators

The BCI’s approach to implementing project goals for under resourced communities is multidisciplinary out of necessity. Community members are demanding equitable representation in research (47). Similarly, these stakeholders are often involved in multiple projects addressing disparities and health equity, which can stretch their capacity. As a component of the BCI collaborative, CAB members receive training to support their responsibilities. To date, members have received training on the Institutional Review Board (IRB) procedures, grantsmanship, research implementation, manuscript preparation, health advocacy, and have mentored researchers in communicating scientific results to lay audiences. Additionally, members serve on a dedicated project outside of the quarterly meetings. For example, three CAB members are currently supporting intervention projects as consultants and are compensated for those activities. These members participate in additional meetings to support implementation activities, and all CAB members contribute to the overall project implementation through recruitment, and evaluation of deliverables during quarterly meetings.

Furthermore, for the researchers that participate in these CBPR activities the competing responsibilities of the academy (i.e., tenure, publications, grantsmanship), and life can be taxing. The current investigators of BCI are from minoritized (e.g., Black, Hispanic, and Asian) backgrounds, and most are early-stage investigators. There are few researchers from minoritized communities that are a part of the academy. Currently, Hispanic, Black, and Asian individuals comprise 6, 7, and 13% of academic faculty, respectively (48). These researchers experience common challenges such as racism, microaggressions, finding meaningful mentor-mentee relationships, and unconscious biases that can contribute to the challenges of engaging in health disparities/equity research (49). We have trained underrepresented students who often gravitate toward projects like BCI because it is applicable to their lived experiences. Some students require additional support with writing, communication, research methods, along with emotional support to successfully complete their internships. Students who worked on BCI projects have received trainings applicable to their personal and professional aspirations and opportunities to contribute to conference abstracts, manuscripts, and presentations to community and academic audiences. One investigator (DTB) uses a group learning model which encourages peer mentoring and fosters a sense of belonging for trainees (50). Various strategies can be employed to support trainees such as improving cultural sensitivity and providing financial resources to effectively train students from minoritized groups (49). Additionally, the responsibility for supporting these students should not rest solely on the few researchers addressing these community issues. The minority taxation on both researchers and community stakeholders is great and calls for a reform (51). The discussions on how to improve these conditions are few, but an important narrative to include in strategies to translate research to community solutions.

## 6 Conclusion

The underlying outcome of translational research should include obtaining the trust of the target community. Yet, the current culture of scientific research does not always support this outcome. Instead, it often widens the knowledge gap between the community and research findings (17). The CBPR method promotes the nurturing of sustainable relationships between academics and the community. As members of the BCI collaborative we understand the need to translate scientific findings into community solutions and engage the Black community as partners to implement interventions toward achieving health equity.

## Data availability statement

The original contributions presented in the study are included in the article/supplementary material, further inquiries can be directed to the corresponding author/s.

## Ethics statement

Written informed consent was obtained from the participant for the publication of this article.

## Author contributions

JT: Investigation, Writing – original draft, Writing – review & editing. AL: Project administration, Writing – original draft, Writing – review & editing. DT: Project administration, Writing – original draft, Writing – review & editing, Conceptualization, Supervision. TF: Writing – review & editing, Conceptualization, Supervision. TT-H: Writing – review & editing, Conceptualization, Supervision. MH: Writing – review & editing, Conceptualization, Supervision. PH: Writing – review & editing, Conceptualization, Supervision. LT: Writing – review & editing, Conceptualization, Investigation, Funding acquisition. DKT-B: Investigation, Writing – review & editing, Conceptualization, Methodology, Project administration, Supervision, Writing – original draft, Resources, Data curation, Funding acquisition, Validation, Visualization.
